# Schnitzler's Disease as an Important Differential Diagnosis of Chronic Recurrent Multifocal Osteomyelitis: A Case Report

**DOI:** 10.1155/2012/318791

**Published:** 2012-08-05

**Authors:** Kathrin Schrödl, Axel Nigg, Marcus Treitl, Michael Flaig, Annette Jansson, Hendrik Schulze-Koops, Christiane Reindl

**Affiliations:** ^1^Division of Pneumology, Medizinische Klinik V, University of Munich, 80336 Munich, Germany; ^2^Division of Rheumatology, Medizinische Klinik IV, University of Munich, 80336 Munich, Germany; ^3^Department for Clinical Radiology, University of Munich, 80336 Munich, Germany; ^4^Dermatology, University of Munich, 80336 Munich, Germany; ^5^Pediatric Clinic, Dr. von Haunersches Kinderspital, University of Munich, 80336 Munich, Germany

## Abstract

*Introduction*. At first sight, chronic recurrent multifocal osteomyelitis (CRMO) and Schnitzler's disease are diagnoses of exclusion and can be similar in their manifestation. *Methods*. In this paper we present the reevaluation of the 13-year-old diagnosis of chronic recurrent osteomyelitis of a 58-year-old man with chronic ostealgia, night sweat, and pruritic urticarial lesions on the extremities and trunk. For further examination, we performed blood analysis, bone and skin biopsies, CT scans, and magnetic resonance imaging. *Results*. Laboratory findings showed increased inflammation parameters. Magnetic resonance imaging (MRI) revealed a diffuse bone marrow infiltration. A bone and skin biopsy showed a sclerotic bone marrow involvement and a superficial dermal and perivascular infiltrate of neutrophils. Based on these findings, the diagnosis of Schnitzler's disease was made. *Conclusion*. Here, we want to present Schnitzler's disease as an important differential diagnosis to CRMO in adults presenting with signs suggestive of CRMO.

## 1. Background

The Schnitzler's syndrome is a rare and underdiagnosed entity which is considered an acquired/late onset auto-inflammatory disease with a median age of onset of 51 years (male > female) [1]. The syndrome is characterized by symptoms of chronic urticarial skin lesions, with histopathological signs of neutrophilic urticarial dermatosis, a monoclonal IgM gammopathy and signs of fever, arthralgia, lymphadenopathy, increased inflammation parameters, and pathologic bone imaging findings [1]. The exact pathogenesis is still unexplained, but successful treatment with the interleukin-1 (IL-1) receptor antagonist, anakinra, suggests a pathogenic overexpression of IL-1 [1, 2]. Here, we report on MRI follow-up investigations of bone marrow infiltration after anakinra treatment.

Chronic recurrent multifocal osteomyelitis (CRMO) is also a rare entity and the most severe form of chronic nonbacterial osteomyelitis [3]. The median age of onset is 10 years. Only 10% of the reported cases start in the early or late adulthood. In the cases of late manifestation, the sternum and diaphysis of the long bones especially the distal femora and the proximal and the distal tibiae are the most affected bones [4, 5]. The main symptoms are pain and swelling of the affected bones, low-grade fever as well as skin disorders normally in form of pustulosis palmoplantaris [6, 7]. Furthermore, histopathological and laboratory findings show a nonspecific chronic osteomyelitis [8]. CRMO is treated with nonsteroidal anti-inflammatory drugs, bisphosphonates, methotrexate, or, lately, by blocking tumor necrosis factor.

## 2. Medical History

In 1998, a 58-year-old man presented himself with chronic ostealgia, night sweat, and pruritic urticarial lesions on the extremities and trunk in a hospital. At that time, bone scintigraphy showed an increased tracer uptake on both sides of the distal femora, the pertrochanteric region as well as the sacroiliac joints. After excluding an infectious cause a bone biopsy of the left proximal femur was conducted. On the basis of the histopathologic findings, the diagnosis of a chronic recurrent multifocal osteomyelitis was made. Accordingly, the patient was treated with nonsteroidal anti-inflammatory drugs with up to 10 g metamizole sodium for the past 13 years.

In February 2011, the patient was referred to our hospital with distinct ostealgia, night sweat, an unclear weight loss of 6 kg in the last 6 month and pruritic urticarial lesions on the extremities and trunk. The patient described a pain level of 9 on a pain scale of 1 to 10.

However, as the chronic recurrent multifocal osteomyelitis is an uncommon diagnosis in elderly people, we reevaluated the previous diagnosis.

To sum up the pathologic findings, the laboratory investigations showed an increased level of C reactive protein (CRP) (6,98 mg/dL), leuocytosis (11,4 G/L), IL-6 elevation (30,6 pg/mL), and minor IgM elevation (596 mg/dL). Radiological findings in the abdominal CT scan showed a marginal enlargement of the intra-abdominal and inguinal lymph nodes. In the T1-weighted images and in the Short Time Inversion Recovery (STIR) images, MRI showed a diffuse bone marrow infiltration of low-to-intermediate signal intensity as well as cortical thickening and periostal bone apposition without surrounding soft tissue reaction in both femora, both tibiae, both clavicles, the left distal fibula, the right os sacrum, both acetabuli, the left os ilii as well as the right os ischii. A conventional bone radiograph of the most painful left femur revealed subtle periosteal reaction along the lateral aspect of the distal metaphysis. A bone biopsy of the left distal femur showed a sclerotic bone marrow involvement, as well as plasma cells, mast cells, sporadic segmented granulocytes and histiocytes, and a lack of suggestive signs of malignancy. The skin biopsy showed a superficial dermal and perivascular infiltrate of neutrophils.

Together, we found no indications for a malignant tumor or inflammatory bowel disease causing the clinical symptoms of systemic inflammation. 

The V198M mutation causing the so-called cryopyrin-associated periodic syndromes in infants could not be found [9]. 

Based on these findings, the diagnosis of Schnitzler's disease was made.

Daily treatment with 100 mg anakinra s.c. was initiated. In the course of treatment, the pruritic urticarial lesions disappeared after 48 hours. After one week, the inflammation parameters normalized, and after four weeks pain therapy was no longer required. Despite all clinical improvements, the 8-week followup of Schnitzler's syndrome by MRI showed only a slight regression of the bone marrow changes and cortical reactions.

## 3. Discussion

CRMO and Schnitzler's syndrome are diagnosed by exclusion. At first sight, both diseases can be similar in their manifestation. However, considering the patient as a whole, the age of onset, the male gender, and the very atypical pruritic urticarial lesions on the trunk led to the correct diagnosis. CRMO is associated with pustulosis palmoplantaris, whereas Schnitzler's disease is associated with neutrophilic dermatitis. Laboratory findings in both syndromes reveal elevated inflammation parameters, but pathognomonic for Schnitzler's disease is an IgM gammopathy. Histopathologic findings of bone biopsies suggest that osteomyelitis is neither characteristic for CRMO nor for Schnitzler's syndrome. The most affected bones in adult CRMO patients as well as in Schnitzler's disease patients are the distal femora and the proximal tibiae [1, 5]. Also, radiological signs of both syndromes seem to be similar, but the radiographs of CRMO-affected bones show lytic destructions demarked by a sclerotic rim. In comparison, lytic lesions are uncommon in Schnitzler's disease [10]. Furthermore, Schnitzler's syndrome radiographs show a slight, linear periostal enlargement of the distal femurs and proximal tibias [10], whereas, in CRMO there is characteristical nonperiosteal elevation or sequestra formation [5]. Magnetic resonance imaging of Schnitzler's disease and active CRMO disease are both described by poorly delimitated bone marrow infiltration with low signal on T1W images and high signal intensity on T2W sequences [11, 12]. The CT imaging in Schnitzler's patients shows enlarged lymph nodes and hepatosplenomegaly, both of which are not typical for CRMO patients.

Hence, Schnitzler's disease is an important differential diagnosis to CRMO in adults presenting with signs suggestive of CRMO.

## References

[1] Lipsker D (2010). The Schnitzler syndrome. *Orphanet Journal of Rare Diseases*.

[2] Kluger N, Gil-Bistes D, Guillot B, Bessis D (2011). Efficacy of anti-interleukin-1 receptor antagonist anakinra (Kineret) in a case of refractory sweet’s syndrome. *Dermatology*.

[3] Girschick HJ, Zimmer C, Klaus G, Darge K, Dick A, Morbach H (2007). Chronic recurrent multifocal osteomyelitis: what is it and how should it be treated?. *Nature Clinical Practice Rheumatology*.

[4] Schilling F (1998). Chronic recurrent multifocal osteomyelitis (CRMO). *RoFo*.

[5] Iyer RS, Thapa MM, Chew FS (2011). Chronic recurrent multifocal osteomyelitis: review. *American Journal of Roentgenology*.

[6] Jurik AG (2004). Chronic recurrent multifocal osteomyelitis. *Seminars in Musculoskeletal Radiology*.

[7] Paller AS, Pachman L, Rich K (1985). Pustulosis palmaris et plantaris: its association with chronic recurrent multifocal osteomyelitis. *Journal of the American Academy of Dermatology*.

[8] Iyer RS, Thapa MM, Chew FS (2011). Imaging of chronic recurrent multifocal osteomyelitis: self-assessment module. *American Journal of Roentgenology*.

[9] Hoffman HM, Mueller JL, Broide DH, Wanderer AA, Kolodner RD (2001). Mutation of a new gene encoding a putative pyrin-like protein causes familial cold autoinflammatory syndrome and Muckle-Wells syndrome. *Nature Genetics*.

[10] Lecompte M, Biais G, Bisson G, Maynard B (1998). Schnitzler’s syndrome. *Skeletal Radiology*.

[11] de Waele S, Lecouvet FE, Malghem J, Jamar F, Lambert M (2000). Schnitzler’s syndrome: an unusual cause of bone pain with suggestive imaging features. *American Journal of Roentgenology*.

[12] Bertrand A, Feydy A, Belmatoug N, Fantin B (2007). Schnitzler’s syndrome: 3-year radiological follow-up. *Skeletal Radiology*.

